# Anticariogenic activity of marine brown algae *Padina boergesenii* and its active components towards *Streptococcus mutans*

**DOI:** 10.3389/fcimb.2024.1458825

**Published:** 2024-11-25

**Authors:** Ravichellam Sangavi, Nambiraman Malligarjunan, Lakkakula Satish, Veerapandian Raja, Shunmugiah Karutha Pandian, Shanmugaraj Gowrishankar

**Affiliations:** ^1^ Department of Biotechnology, Alagappa University, Karaikudi, India; ^2^ Applied Phycology and Biotechnology Division, Marine Algal Research Station, CSIR-Central Salt & Marine Chemical Research Institute, Mandapam, India; ^3^ Center of Emphasis in Infectious Diseases, Department of Molecular and Translational Medicine, Paul L. Foster School of Medicine, Texas Tech University Health Sciences Center, El Paso, TX, United States

**Keywords:** biofilm, dental caries, MEPB, GC-MS/MS, *S. mutans*, virulence

## Abstract

*Streptococcus mutans* is a well-recognized bacterium that plays a predominant role in the progression of dental caries. Its pathogenicity is linked to several key characteristics, including the ability to produce organic acids (acidogenicity), thrive in low pH environments (aciduricity), synthesize exopolysaccharides (EPS) *via* glucosyltransferases, and form retentive biofilms. The treatment of dental caries with conventional antibiotics is often ineffective due to the bacterium’s capacity to form recalcitrant biofilms. To address these challenges, strategies that specifically target the pathogen’s virulence without affecting its viability have emerged as promising alternatives. In this context, we investigated the anticariogenic properties of the methanolic extract of *Padina boergesenii* (MEPB). MEPB demonstrated substantial, dose-dependent antibiofilm activity, with a maximum inhibition of 93% at 128 μg/mL, without compromising the viability of *S. mutans*. Anti-virulence assays using sub-MIC (minimum inhibitory concentration) levels of MEPB showed significant reductions in key virulence factors: 75% reduction in sucrose-dependent adherence, 65% reduction in sucrose-independent adherence, along with notable decreases in acid production, acid tolerance, and water-insoluble (85%) and water-soluble (52%) glucan synthesis. Additionally, MEPB significantly reduced cell surface hydrophobicity (55%) and extracellular DNA (eDNA) production (64%). qPCR analysis corroborated these *in vitro* findings, revealing that MEPB suppresses the expression of genes involved in *S. mutans* virulence, particularly genes related to EPS synthesis (*gtfB*, *gtfC* & *gtfD*) biofilm formation(*gbpB* & *gbpC*) and two-component regulatory system (*vicR)* were downregulated. Toxicity testing on human buccal epithelial cells confirmed the non-toxic nature of MEPB, suggesting its safety for potential therapeutic use. Furthermore, GC-MS/MS analysis identified palmitic acid, myristic acid, and stearic acid as the major active constituents of the MEPB extract. Subsequent biofilm inhibitory assays confirmed the potent antibiofilm efficacy of these compounds: palmitic acid (85%), myristic acid (72%) and stearic acid (83%). In conclusion, this study identifies *P. boergesenii* and its active biomolecules as potential anticariogenic agents, offering an alternative approach to combat dental caries by targeting bacterial virulence mechanisms rather than viability.

## 1 Introduction

Dental caries is a widespread, biofilm mediated and diet-modulated disease-affecting individuals worldwide regardless of their age, gender or socioeconomic status (Uribe et al., 2021). Its prevalence not only diminishes the overall quality of life but also exerts psychological impact on the well-being and performance of affected individuals (Teshome et al., 2021; Nath et al., 2023). As per the World Health Organization (WHO) report of 2022, untreated dental caries stands as the most prevalent global condition, influencing approximately 2.5 billion individuals. Despite substantial advancements in technology and a growing awareness in oral health care, the incidence rate of dental caries remains astonishingly high, emphasizing this infection as significant global public health concern (Teshome et al., 2021).

Although polymicrobial consortia play a role in driving caries development, *S. mutans* is considered the primary pathogen responsible for the initiation and progression of dental caries. Unlike most pathogens that exhibit classic virulence factors, *S. mutans* possesses unique virulence traits primarily associated with carbohydrate metabolism. These traits include: (i) the production of organic acids *via* glycolysis, (ii) the ability to survive in low pH environments (aciduricity) (Moye et al., 2014), (iii) synthesize of extracellular polysaccharides (EPS) by utilizing sucrose and (iv) adherence to glucan coated tooth surface (Bowen and Koo, 2011). These attributes facilitate *S. mutans* to effectively colonize the oral cavity, resulting in the formation of highly cariogenic dental plaques.

Initial colonization and subsequent *S. mutans’* biofilm formation is achieved mainly through two mechanisms: sucrose -dependent and -independent mechanism. The sucrose- dependent mechanism primarily relies on glycosyltransferase (Gtf-B, C and -D) that are crucial for synthesizing glucans from sucrose. In specific, GtfB fosters a water-insoluble glucans that are enriched with α (1-3)-linkages, whereas, GtfC synthesizes a mixture of soluble and insoluble glucans that are abundant in α (1-6)-linkages and GtfD predominantly synthesizes water soluble glucans (Nijampatnam et al., 2018). Prior research has demonstrated that the glucans generated by GtfB and GtfC are crucial for initial bacterial adhesion, biofilm formation and maintaining the structural integrity of extracellular matrix. Meanwhile, the glucans synthesized by GtfD serve not only as a primer for GtfB but also as a nutrient source for *S. mutans* and other oral bacteria (Falsetta et al., 2012). Furthermore, earlier research in this area has indicated that *gtfB* and *gtfC* gene deletion significantly hampers formation of both microcolonies and biofilms in *S. mutans* (Fears et al., 2015). Additionally, *S. mutans* possess several glucan binding proteins (Gbps) namely, Gbp (A, B, C and D) that enhance bacterial adhesion to glucans and also promote the co-aggregation thereby pave a way for plaque formation (Duque et al., 2011).

*S. mutans* primarily depends on fermenting dietary sugars to produce energy for its growth and survival, since it does not possess a complete tricarboxylic acid cycle or respiratory chain (Ajdić et al., 2002). Once sugars are internalized into the cytoplasm, they undergo phosphorylation and are metabolized into organic acids through glycolysis, leading to a significant drop in pH within oral biofilms, decreasing from a neutral pH to levels well below 5 (Moye et al., 2014). *S. mutans* survives in acidic environments by regulating pH levels across its cell membrane and maintaining an alkaline environment within the cell rather than the surroundings. This is accomplished by three mechanisms: the overexpression of the F1F0-ATPase system, membrane fatty acid synthesis (FabM), and branched-chain amino acid biosynthesis (Klein et al., 2012). Therefore, *S. mutans* with all its virulence traits persist as a cariogenic pathogen, leads to the enamel dissolution and eventually tooth loss (Falsetta et al., 2012). Further, a plethora of studies have demonstrated that mutants of *S. mutans* deficient in these virulence factors are comparatively exhibits less cariogenic potential as well as become more vulnerable to various environmental stresses than wild-type strain (Hasan et al., 2014). Therefore, therapeutic strategies targeting the *S. mutans* virulence factors, hold significant promise for preventing caries and maintaining oral health.

While there are numerous antibacterial agents recognized for their ability to decrease dental biofilm formation, only a few have demonstrated significant effectiveness such as fluoride and chlorhexidine. This is primarily attributed to the resilience conferred by *S. mutans* biofilms, which make them resistant to mechanical abrasions and antimicrobial agent treatments. Additionally, excessive fluoride application is associated with adverse effects such as fluorosis, limiting its widespread application (Hassan et al., 2015). Furthermore, chlorhexidine, a widely used standard anti-plaque agent, has been reported to have genotoxic properties (Ribeiro et al., 2004).

In this pursuit, currently there is considerable interest in seeking approaches that effectively prevent or treat infections associated with biofilms. Hence, development novel anticariogenic agents those specifically suppress or inhibit the *S. mutans’* virulence rather than solely focusing on growth. This precise targeting is a feasible strategy to prevent dental caries without promoting the emergence of bacterial resistance by reducing development selective pressure, while preserving natural microflora of the oral cavity.

Marine ecosystems still largely constitute an untapped resource rich in structurally unique and biologically active compounds. Seaweeds have been used in traditional medicine for centuries due to their therapeutic potential in treating various diseases (Pérez et al., 2016). Research has demonstrated that secondary and some primary metabolites derived from green, brown, and red marine algae exhibit activities such as anti-quorum sensing (Tang et al., 2020), antimicrobial (Pérez et al., 2016), anti-inflammatory (Ramasubbu et al., 2023), anticancer (Balaji et al., 2023) and antioxidant (Moussavou et al., 2014) activities. In general, algae belonging to the phaeophyta are more effective against biofilm-forming organisms compared to green algae (Makhlof et al., 2024).

In particular, *Padina boergesenii*, a brown seaweed from the Dictyotaceae family, commonly found along the southeast coast of India in the Gulf of Mannar, is noted for its high levels of phenolics and fatty acids (Kumar and Sudha, 2012). Previous studies have demonstrated the antimicrobial and antibiofilm activities of *P. boergesenii* against several clinically significant pathogens such as *Staphylococcus aureus* (Sethupathy et al., 2017), *Pseudomonas aeruginosa* (Ragunath and Ramasubramanian, 2022) and *Candida albicans* (Arumugam et al., 2019). Despite this wealth of knowledge, there has been a notable gap in research concerning their promising anticariogenic potency against *S. mutans*. The present study addresses this gap and represents a significant advancement by providing comprehensive evidence of the remarkable anticariogenic efficacy of marine macroalgae *P. boergesenii* and its active compounds against *S. mutans*.

## 2 Methods

### 2.1 Ethical statement

To perform toxicity analysis, Human Buccal Epithelial cells (HBECs) utilized that are obtained from consenting healthy volunteers. The experimental protocol involving HBECs collection was appraised and permitted by the Institutional Ethical Committee of Alagappa University, Karaikudi (IEC Ref No: IEC/AU/2018/5). Further, HBECs collection procedure was performed according to established guidelines and regulations.

### 2.2 Collection and preparation of seaweed extract

The brown seaweed *P. boergesenii* was harvested from the Durgapur jetty (13° 16’ 10.002’’ N, 93° 2’ 26.0808’’ E) located in Ariel Bay village, North Andaman. After collection, the seaweed was rinsed with clean seawater followed by distilled water to eliminate any debris and contaminants. The cleaned seaweed was then transported by placing them on ice to the laboratory. Upon arrival, seaweed was sliced into pieces, shade-dried before being ground into a powder fine. For methanolic extraction, 10 g of the seaweed powder was added to 300 mL of methanol in a conical flask, maintaining a ratio of 1:30 w/v. This preparation was placed in the shaking incubator at room temperature for a period of 3 days. Following this extraction period, the preparation was spun at 1000 rpm for 12 min and the obtained supernatant was evaporated using vacuum evaporator (Kalasariya et al., 2023). The resulting dried residues were stored at 4°C until needed. For subsequent *in vitro* assays, the crude extract was dissolved in methanol to achieve a concentration of 100 mg/mL.

### 2.3 Strains and culture conditions

The test organism, *S. mutans* UA159 was obtained from HiMedia, India. *S. mutans* UA159 was cultured and maintained in Todd Hewitt Broth (THB) medium. For routine culturing and *in vitro* assays THB medium containing 1% yeast extract and 1% sucrose (THYS) was used.

### 2.4 Minimum inhibitory concentration

The MIC of the methanolic extract of *P. boergesenii* (MEPB) on *S. mutans* was measured using the broth microdilution technique described by Jothi et al. (2021). In this experiment, THYS medium was inoculated with 5.0×10^-5^ CFU/mL of *S. mutans* in a microtiter plate at different MEPB concentrations ranging from 2 to 1024 µg/mL. THYS medium containing 5.0×10^-5^ CFU/mL of *S. mutans* served as the control, while THYS medium with 10 µL of methanol and 5.0×10^-5^ CFU/mL of *S. mutans* was used as the vehicle control to assess the effect of the solvent on *S. mutans’* growth. The MIC was calculated by measuring the cells at OD_600nm_ following a 24 h anaerobic incubation period at 37°C. MIC was identified as the least concentration of MEPB that totally inhibited visible growth of bacteria.

### 2.5 Growth curve assay

The growth inhibitory effect of MEPB at 32, 64 & 128 µg/mL on the growth of *S. mutans* was examined using the growth curve analysis (Selvaraj et al., 2020). In the assay, *S. mutans* aliquots overnight culture was diluted to attain 5×10^-5^ CFU/mL in THYS broth. Sub-MICs of MEPB were subsequently added to these cultures, and they were then incubated anaerobically for 24 h at 37°C. THYS medium containing 5.0×10^-5^ CFU/mL of *S. mutans* served as the control. Furthermore, using a spectrophotometer set to OD_600nm_, the *S. mutans* growth was measured for each for every one over a 24 h period.

### 2.6 Assessment of cellular viability

To evaluate the viability of cells in both control as well as MEPB sub-MIC treated samples, the alamar blue method was performed (Priya et al., 2021). Initially, cells from both untreated and treated samples were collected, rinsed and resuspended in fresh PBS. Next, alamar blue was added to the suspended cells and incubated for 1 h at 37°C in the dark. A solution of PBS containing alamar blue have been used as a blank. The supernatant was recovered by centrifuging the samples for 15 min at 6000 rpm after the incubation period. Further, the fluorescence intensity was read at OD_560nm_ and OD_590nm_ for emission and excitation, respectively.

### 2.7 Biofilm inhibitory assay

The efficacy of MEPB against *S. mutans* biofilm formation was evaluated through adopting the methodology described by Valliammai et al. (2019). Sub-MICs of MEPB were added to THYS medium containing bacterial suspensions at 5×10^-5^ CFU/mL in the 24-well polystyrene plate. THYS medium containing 5×10^-5^ CFU/mL of *S. mutans* was used as the control. After incubation, the cells were carefully washed with PBS to eliminate non-adherent cells and subsequently 0.4% of crystal violet was appended to the residual biofilm. Further, 15% of glacial acetic acid was introduced to the wells and read at OD_595nm_. The control wells contained medium without MEPB, whereas the blank wells contained medium and MEPB.

### 2.8 Light microscopy

To visualize the *S. mutans* biofilm formation, glass slides measuring 1 × 1 cm were used. Biofilms were allowed to develop on these slides with and without presence of MEPB at sub-MICs. Following incubation time for 24 h, the slides with the biofilms were carefully removed, washed with PBS and air-dried. Subsequently, the slides were incubated with crystal violet (0.4%) for 15 min. Further, slides were rinsed with distilled H_2_O and air-dried to remove any remaining unbound stain. The CV-stained slides were then inspected under light microscope (Nikon Eclipse Ts2R, Japan) (Valliammai et al., 2019).

### 2.9 LIVE/DEAD analysis of biofilm

The biofilm of *S. mutans* were cultivated on glass slides with or without MEPB at sub-MICs. 0.12% chlorhexidine was served as positive control. After incubation for 24 h at 37°C, the slides underwent triple washing with sterile PBS. Subsequently, they were stained with the acridine orange (AO) & propidium iodide (PI) and left in dark for 15 min. After staining, the excess dye was removed by washing the glass slides with PBS. Following air-drying, the stained cells were then inspected using CLSM (Zeiss LSM-710; Carl Zeiss, Oberkochen, Germany) (Jothi et al., 2021).

### 2.10 Effect of MEPB on adherence

To investigate the potential of MEPB to inhibit *S. mutans* adherence to tooth surfaces, an adherence assay was conducted following the method by Viszwapriya et al. (2017). Initially, *S. mutans* was cultured in THYS medium with and without MEPB in glass tubes tilted at a 30° angle, and then incubated for 24 h at 37°C. After incubation the non-adherent or free-floating were cells removed, the adherent or cells bound on glass surface were carefully removed by rinsing the surface of the glass tubes with using a pipette, and their OD was recorded at 600_nm_ with a spectrophotometer. The percentage of adhesion (%) is computed as (OD of adherent cells/OD of total cells) × 100.

### 2.11 Cell surface hydrophobicity

To assess the impact of MEPB on *S. mutans* surface hydrophobicity, experiments were conducted based on the methodology by Jothi et al. (2021). Initially, *S. mutans* was incubated for 24 h with and without MEPB at sub-MICs at 37°C. Following incubation, the cells were collected, washed twice and resuspended in 0.85% sterile saline, adjusting the suspension to an OD of 1.0. Later, the cell suspension was mixed with 1 mL of toluene and the tubes were vortexed uniformly for 5 min. Afterwards, the tubes were allowed to stand at room temperature for 10 min to facilitate phase separation between aqueous phase and toluene. After separation, the aqueous phase OD was read at 600_nm_ using a spectrophotometer. The relative hydrophobicity index was determined using the formula: [1 − (OD_600nm_ after vortexing/OD_600nm_ before vortexing) × 100.

### 2.12 Autoaggregation assay

To analyze the impact of MEPB on *S. mutans* autoaggregation property, bacterial cultures were grown with and without sub-MICs of MEPB. Post incubation with for 24 h at 37°C, the cells were retrieved by centrifugation and dissolved in 1X PBS. These suspensions were further allowed to stand undisturbed and observed for aggregation after 30 min period. Afterward, 200 µl samples were taken from the upper layer of each suspension, and their OD at 600_nm_ was measured to assess autoaggregation (Sorroche et al., 2012).


$$\%\text{ aggregation}=\left[\left(OD_{initial}-OD_{final}\right)/OD_{initial}\right)]\times 100$$

### 2.13 Estimation of glucans production

To quantify both water-soluble and -insoluble glucans synthesis, the phenol/sulfuric acid method was employed following Gowrishankar et al. (2014). Initially, *S. mutans* was added to the THYS broth supplemented with and without MEPB at sub-MICs and then incubated at 37°C for 24 h. After incubation, the samples were subjected centrifugation to collect the supernatant encompassing extracellular components. To isolate water-soluble glucans, the collected supernatant was precipitated with ethanol. The remaining cell pellets were subsequently resuspended in 1 M NaOH. Further, the mixture was centrifuged and the obtained supernatant was precipitated with ethanol to acquire water-insoluble glucans.

### 2.14 Acid production

The influence of MEPB on the *S. mutans* glycolytic efficiency was evaluated using a pH drop assay, as mentioned by Goc et al. (2019). In brief, *S. mutans* was harvested by centrifugation, then washed and re-dissolved in a salt solution (50 mM KCl &1 mM MgCl_2_). To the cells, sub-MICs of MEPB were added. Salt solution containing *S. mutans* alone acted as control. Following that, the suspension’s pH was adjusted to 7.2 by adding the required volume of 0.1 M KOH and finally, 1% (w/v) of glucose was added. *S. mutans* glycolytic activity, as indicated by a drop in pH, was measured at 15-min intervals throughout a 120-min period.

### 2.15 Acid tolerance

To investigate MEPB efficiency on the capability of *S. mutans* to withstand and survive under acidic conditions (pH 5.0) was evaluated following the He et al. (2019). The overnight grown bacterial cells were retrieved through centrifugation and re-dissolved in THYS medium with pH 5.0. To the medium with varying concentrations of MEPB sub-MICs was added. Following the incubation at 37°C for 2 h, the treated and untreated bacterial cells were subjected to serial dilution and plated on THYS agar to determine the count of survived cells.

### 2.16 eDNA extraction

To extract eDNA, 5 mL of THYS medium were inoculated with 1% *S. mutans* in 6-well plate, both with and without MEPB at sub-MICs. The planktonic cells were cautiously removed without damaging the biofilm cells after 24 h incubation. The biofilm was then exposed to 1 mL of TE buffer (10 mM Tris & 0.5 mM EDTA). After carefully scraping off the cells and biofilm matrix, the mixture was transferred to fresh tube and vortexed for 15 mins to release the eDNA followed by centrifugation to collect the liberated eDNA. To visualize the eDNA, 15 µL of the collected supernatant was applied onto a one percentage agarose gel, stained with ethidium bromide and observed. Additionally, eDNA extracted from control and MEPB-treated samples were quantified using a Bionano Spectrophotometer (Shimadzu, Kyoto, Japan) (Yu et al., 2024).

### 2.17 H_2_O_2_ sensitivity

The survival ability of *S. mutans* was analyzed using H_2_O_2_ sensitivity assay. In brief, control and MEPB treated *S. mutans* cells were spun at 8000 rpm for 10 min and the resulting pellets were dissolved in 1 ml PBS. The cells were then treated with 0.2% of H_2_O_2_ followed by incubation at 37°C for 2 h. After incubation, the samples were then spread on THYS agar and left overnight to assess bacterial colonies (Priya et al., 2021).

### 2.18 Quantitative real-time PCR analysis

To investigate the impact of MEPB on the *S. mutans* virulence-associated genes namely, *gtfB, gtfC, gtfD, vicR, gbpC* and *gbpB*, quantitative real-time PCR (qPCR) analysis was executed following a protocol described by Gowrishankar et al. (2014). Total RNA was isolated from both control and MEPB-treated *S. mutans* cells employing the TRIzol technique. The extracted RNA was then processed with DNase I to remove any contaminating DNA. Next, cDNA was synthesized using an Applied Biosystems high-capacity cDNA reverse transcription kit. Next, qPCR was carried out using Power SYBR green PCR master mix (Applied Biosystems) on a 7500 thermal cycler sequence detection system (Applied Biosystems Inc., Foster City, CA, USA), in compliance with the manufacturer’s instructions. In order to measure the levels of gene expression, the cycle threshold (CT) values of virulence genes were normalized to a housekeeping gene (16S rRNA) using the 2^−ΔΔCT^ technique. The primers used in qPCR is listed in **Table 1**.

**Table 1:** Primers used in the qPCR analysis.

Gene	Description	Primer sequence (5’ – 3’)	
		Forward	Reverse
*vicR*	Two-component regulatory system	TGACACGATTACAGCCTTTGATG	CGTCTAGTTCTGGTAACATTAAGTCCAATA
*gtfB*	Water insoluble glucan synthesis	AAAGCAACGGATACAGGGGA	CTCTGTCATTGGTGTAGCGC
*gtfC*	Water soluble and insoluble glucan synthesis	GGTTTAACGTCAAAATTAGCTGTATTAGC	CTCAACCAACCGCCACTGTT
*gtfD*	Water soluble glucan synthesis	GAAGTATGGCGGTGCTTTCC	ATAACCAACACCACGGCCTA
*gbpB*	Glucan binding protein	ATGGCGGTTATGGACACGTT	TTTGGCCACCTTGAACACCT
*gbpC*	Glucan binding protein	TCTGGTTTTTCTGGCGGTGT	GTCAATGCTGATGGAACGCC
*16S rRNA*	House-keeping gene	ACTCCTACGGGAGGCAGCAG	ATTACCGCGGCTGCTGG

### 2.19 Toxicological evaluation on human buccal epithelial cells

In order to assess the safety profile of MEPB, HBECs were used as described by Souza et al. (2018). Healthy volunteers with good oral hygiene were chosen for the study. The cotton swab was used to collect HBECs by gently swabbing the inner area of the cheeks. The cotton swabs were then immersed in PBS, collected cells were mixed together and centrifuged at 6000 rpm for 10 min. The resulting pellet was washed three times with PBS to remove any debris. After removing the debris with PBS, the HBECs suspension was prepared to a final 5.0 × 10^-5^ cells/mL. Next, the HBECs were exposed to MEPB for 20 min at 37°C to determine any potential toxic effects. 10% of Hydrogen peroxide serves as control. Post incubation, cells were stained with a mixture of AO and PI in a 1:1 ratio and incubated in the dark for 15 min. The live and dead cells of control and MEPB-treated HBECs were then observed using fluorescence microscope (Nikon Eclipse Ts2R, Japan).

### 2.20 Identification of compound through GC-MS/MS

The components of MEPB were analyzed using a SHIMADZU GCMS138 QP2010 plus system. The analysis employed an Rxi^®^-5ms gas chromatograph column with dimensions of 0.25 mm and 0.25 µm internal diameter and film thickness, respectively. This column was connected to a mass spectrometer detector and comprised 95% and 5% dimethylpolysiloxane and diphenyl, respectively. The column, measuring 30 meters in length, was set with an initial oven temperature of 150°C for 1 min. The temperature was raised to 180°C at a rate of 10°C/min and held for 4 min and then consequently raised to 300°C at 15°C/min, with a final hold of 17 min. Helium was used as the carrier gas and injected with a flow rate of 1 µL/min constantly. Moreover 2 µL volume was injected to employ the split ratio of 1:10. Both the ionization and interface temperatures were programmed at 200°C and subsequently the scan range was set between 40 and 450 atomic mass units. Peak identification and analysis were carried out using the NIST library (Gowrishankar et al., 2014).

### 2.21 Antibacterial and antibiofilm activity of identified compounds in alone and combination

The effect of active compounds (palmitic acid, myristic acid and stearic acid) against the *S. mutans* planktonic cells viability and biofilm forming ability was assessed. The procedures were followed as described in 2.4. and 2.7 for MIC and BIC. The active compounds such as palmitic acid, myristic acid and stearic acid (TCI chemicals, Japan) was dissolved in methanol with stock concentration of 100 mg/mL.

### 2.22 Statistical analysis

Experimentations were conducted in biological triplicates with at least two repetitions, and findings were presented as mean ± SD. One-way analysis of variance (ANOVA) was used to compare control and treatment samples, followed by Dunnett’s *post hoc* test, which was carried out employing SPSS statistical software version 17.0. Significance was defined as *p ≤*0.05, *p* < 0.01, or non-significant. All of the statistical data was transformed into a graph by GraphPad Prism v9.5.1.

## 3 Results

### 3.1 Antibacterial efficacy of MEPB against *S. mutans*

**Figure 1:**
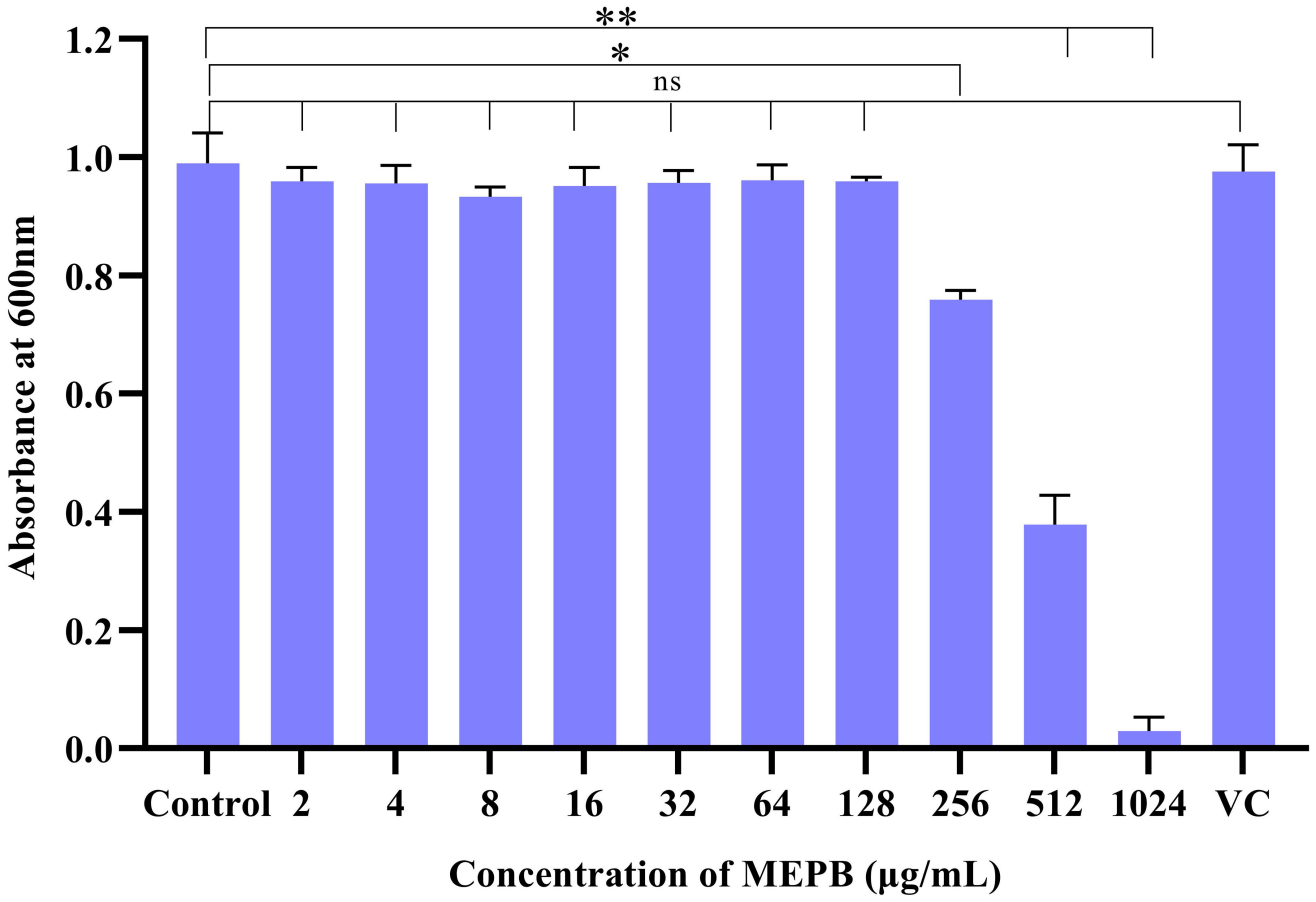
The efficacy of MEPB on the growth *S. mutans*. *S. mutans*’ growth was dramatically suppressed by MEPB in a concentration-dependent manner. MEPB showed evident growth inhibition at 1024 µg/mL. VC- Vehicle Control. All the experiments were conducted triplicate; error bars represent means ± SD. “*”, “**” and “ns” indicates *p* ≤0.05, *p* <0.01, and non-significant, respectively.

The antibacterial effect of MEPB on *S. mutans* determined using a broth microdilution assay. The results showed that MEPB exerted antibacterial effects at concentrations of 256, 512, and 1024 μg/mL. Specifically, at 256 μg/mL, MEPB reduced the *S. mutans* growth by 20%, while at 512 μg/mL, it reduced growth by 60%. MEPB suppressed *S. mutans* growth completely at 1024 μg/mL, and therefore 1024 μg/mL was known to be the MIC. Further, methanol up to 10 μL did not exhibit any growth inhibitory effect on *S. mutans*. This indicates that the observed growth inhibition is solely due to MEPB and not the methanol used as a solvent (**Figure 1**).

### 3.2 Effect of MEPB on the *S. mutans* growth

**Figure 2:**
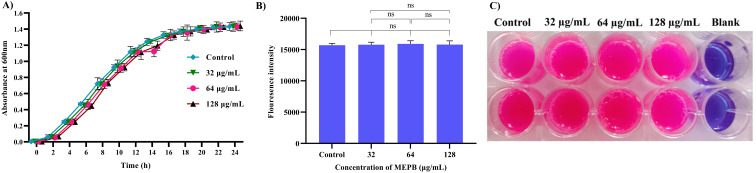
Impact of planktonic growth of *S. mutans* at sub-MICs (32-128 µg/mL) with and without the MEPB. **(A)** The growth curve analysis clearly depicts the non-significant changes in growth of *S. mutans* in the absence and presence of MEPB **(B)** Assessment of metabolic viability in the presence and absence of MEPB. There was no significant growth decrease reported at sub-MICs. The data is provided as mean ± SD (n = 3), with the error bar representing the standard deviation. The non-significant is represented by the symbol “ns”. **(C)** Representative plate image of metabolic viability assay.

Unlike antibiotics, which exerts direct bactericidal effects, antivirulence drugs aim to mitigate the production of virulence factors without interfering with bacterial growth. To confirm the non-bactericidal activity of MEPB at sub-MIC levels (32, 64 and 128 µg/mL), a growth curve analysis and metabolic viability assay were performed, since this sub-MICs were utilized in subsequent virulence assays. Growth curve analysis findings (**Figure 2A**) showed no statistically significant changes in the growth patterns at any time point between the MEPB-treated *S. mutans* and the untreated control. This finding was further supported by the alamar blue-based metabolic viability experiment. In this experiment, the electron transport mechanism of live cells reduces resazurin to resorufin, contributing to a color shift from blue to pink. The intensity of the pink color corresponds directly to the quantity of live cells. Cells treated to MEPB at 32, 64, and 128 µg/mL developed a pink color comparable to the control cells. These results confirm that MEPB does not exhibit bactericidal activity against *S. mutans* at sub-MIC concentrations, supporting its potential as an antivirulence agent that targets virulence factor production without affecting bacterial growth.

### 3.3 Antibiofilm activity of MEPB against *S. mutans* biofilms

**Figure 3:**
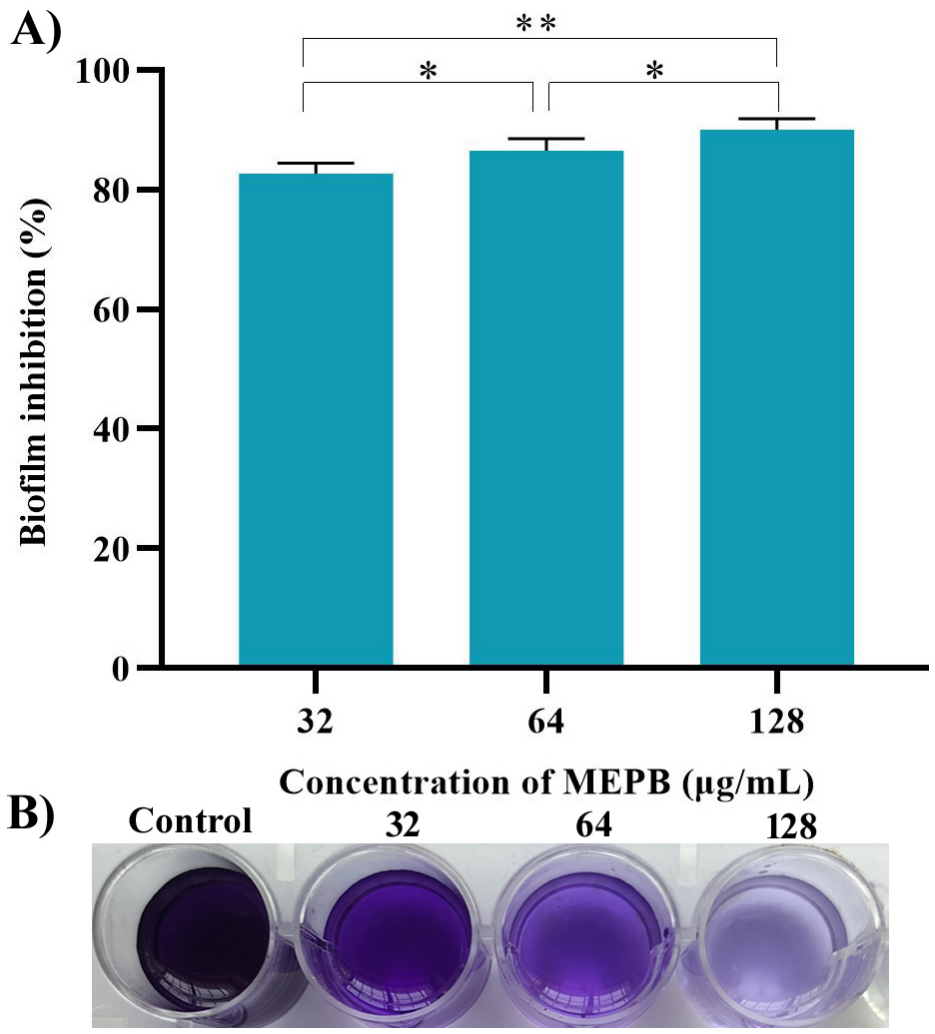
MEPB’s influence on biofilm development in *S. mutans*. **(A)** Spectrophotometric quantification reveals a concentration-dependent decrease in biofilm biomass of *S. mutans* under MEPB treatment. **(B)** A representative plate image showing biofilm inhibition. The data is reported as mean (n = 3), with error bars representing standard deviation. The symbols “*”, “**” indicates statistical significance at *p*≤0.05 and *p*<0.01, respectively.

A crystal violet-based biofilm biomass quantification assay demonstrated that MEPB effectively suppressed *S. mutans* biofilm formation on polystyrene plate in a dose-dependent manner. Remarkably, 28 µg/mL of MEPB reduced *S. mutans* biofilm mass by up to 93% (*p* < 0.01) (**Figure 3**). MEPB, even at the lowest concentration (32 µg/mL), effectively inhibited more than 75% of the biofilm formation (*p ≤*0.05) without adversely affecting the basic cellular metabolism of *S. mutans*, as confirmed by growth curve analysis and a metabolic viability assay (**Figure 2**).

### 3.4 Biofilm structure visualization

**Figure 4:**
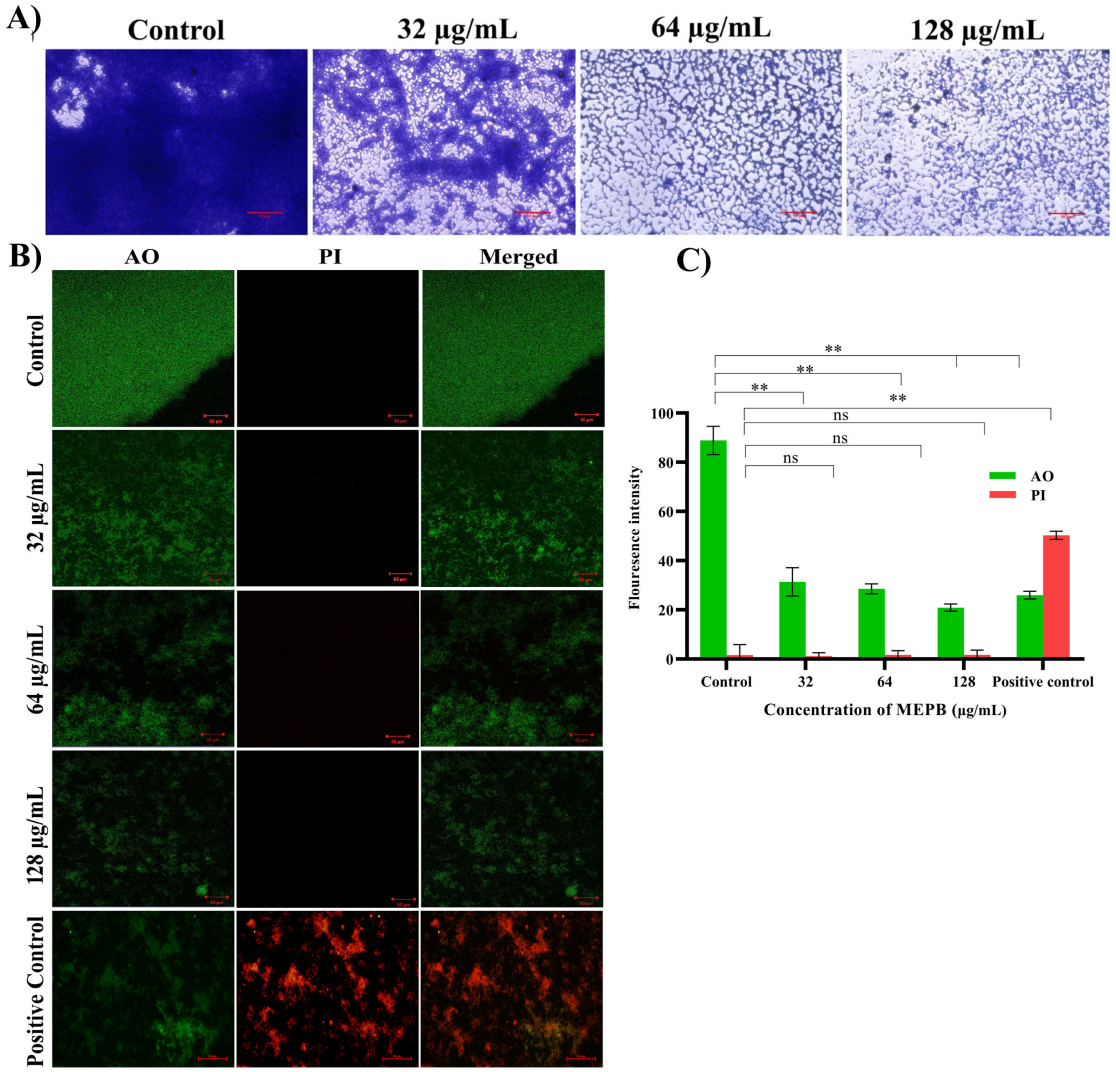
Microscopic observation of *S. mutans* biofilms produced on the glass surface after 24 h of incubation. **(A)** Light micrographs of control samples revealed tightly packed multifaceted biofilm structures. While the micrograph of MEPB-treated samples showed decreased and scattered biofilm architecture. **(B)** Live/dead analysis Images of MEPB-treated and untreated control samples under a microscope (magnification: ×200, scale bar 50 µm) reveal a greater number of live cells than dead cells. **(C)** The bar graph depicts the fluorescence intensity of the AO and PI. Standard deviation and statistical significance (*p* ≤0.05, *p* <0.01 and “non-significant” respectively) are shown by error bars and asterisks “*” “**” and “ns”.

Biofilms formed after 24 h of incubation with varying sub-MICs of MEPB were examined using a light microscope (**Figure 4A**). In the absence of MEPB, the biofilms exhibited uniform surface coverage and a dense structure. However, treatment with 128 µg/mL of MEPB resulted in highly dispersed and visibly loose biofilms, significantly reducing the surface area covered and leading to a substantial decrease in biofilm biomass. Similarly, significant biofilm inhibition was observed at 32 and 64 µg/mL concentrations. Overall, microscopic examinations revealed that MEPB attenuates the biofilm forming ability of *S. mutans* in a dose-dependent fashion.

### 3.5 Non-bactericidal activity of MEPB at sub-MICs on *S. mutans* biofilm

To accurately assess the *S. mutans* biofilm cells viability when exposed to MEPB, a LIVE/DEAD analysis were conducted. Acridine orange (AO), a cell-permeable dye, stains both living and dead cells, whereas propidium iodide (PI) exclusively stains dead cells. According to the CLSM, the presence of higher number of live cells than dead cells was observed in control samples. Similarly, cells treated with MEPB at concentrations 32, 64, and 128 µg/mL also showed a greater number of living cells and fewer dead cells (**Figure 4B**). Further, the results were ascertained through measuring the fluorescence intensity of the AO and PI. The results signify that, the presence of dead biofilm cells in MEPB treated was very similar to that of untreated control. This assay further confirmed that MEPB reduced *S. mutans* biofilm formation without affecting its growth.

### 3.6 Anti-adherence efficacy of MEPB against *S. mutans*

**Figure 5:**
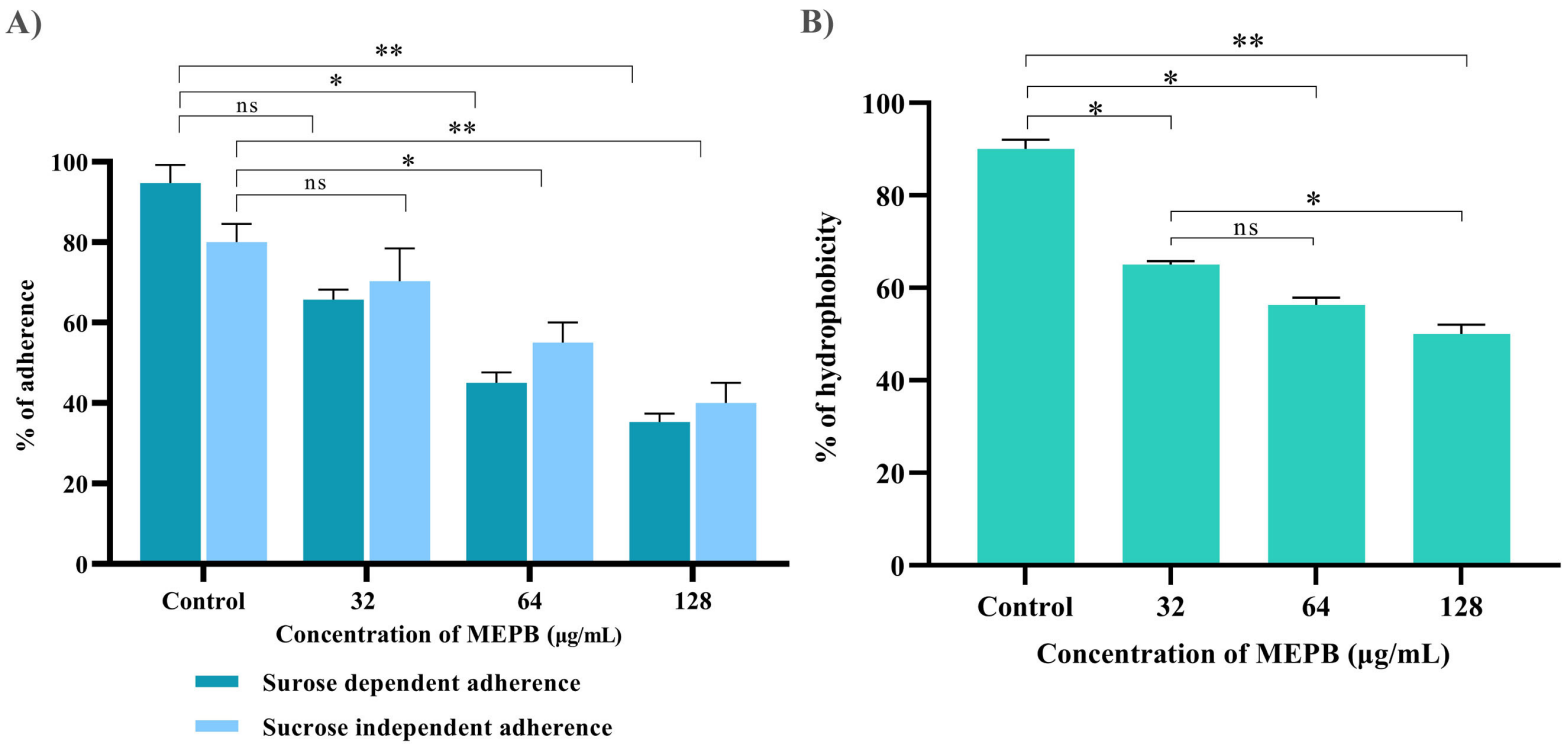
Influence of MEPB on the adherence and hydrophobicity of *S. mutans*. **(A)** The bar graph depicts the percentage of adherence to MEPB treatment at absorbance 600_nm_. MEPB, at the utilized concentration dramatically decreased both sucrose-dependent and independent adherence. **(B)** The bar graph shows the percentage of hydrophobicity reduced after MEPB treatment compared to the untreated control. The asterisks “*,” “**,” and “ns,” together with error bars, indicate the standard deviation and statistical significance (*p* ≤0.05, *p* <0.01, and “non-significant,” respectively).

Anti-adherence activity of MEPB on *S. mutans* was assessed using various sub-MICs, as shown in **Figure 5A**. With the increasing concentrations of MEPB, the sucrose-dependent and sucrose-independent adhesion was decreased. The results show that the 128 µg/mL of MEPB considerably reduced both sucrose-dependent and -independent adherence by 75% (*p* < 0.01) & 65% (*p <*0.01), respectively (**Figure 5A**). Interestingly, MEPB decreased sucrose-dependent adherence by 25% even at the lowest dose (32 µg/mL), suggesting its potency in reducing the adherence even in the presence of sucrose. These findings indicate that MEPB substantially lowers *S. mutans* adherence in both sucrose-dependent and -independent conditions, with a greater effect on sucrose-dependent adherence.

### 3.7 Effect of MEPB on *S. mutans* hydrophobicity

Bacterial adhesion to the enamel is largely dependent on the hydrophobic nature of the cell surface. This hydrophobicity was evaluated by calculating the number of cells of adhered to hydrocarbons. The results indicated that untreated *S. mutans* cells had a higher affinity for toluene, which was reduced in MEPB-treated cells in a concentration-dependent manner (**Figure 5B**). Moreover, 128 µg/mL of MEPB, reduced the hydrophobicity of *S. mutans* by nearly 55% (*p <*0.01) in comparison with untreated control. Further, the concentration 32 and 64 µg/mL also decreased the hydrophobicity to 35 and 45% (*p ≤*0.05). The results revealed that MEPB significantly reduces the *S. mutans* hydrophobicity, potentially impairing its capacity to form biofilms.

### 3.8 Effect of MEPB on autoaggregation of *S. mutans*

**Figure 6:**
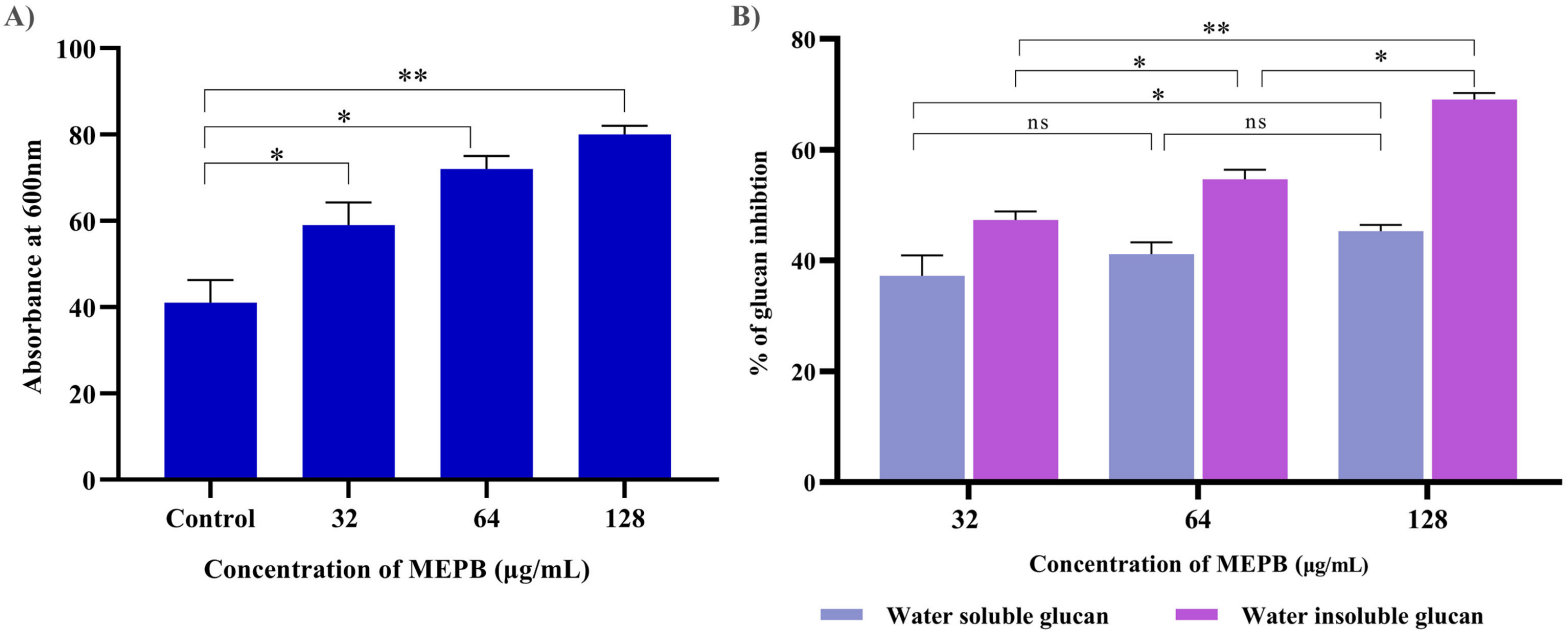
Antivirulence activity of MEPB on *S. mutans*. **(A)** The bar graph depicts the proportion of autoaggregation after MEPB treatment at OD600_nm_. MEPB, at the sub-MICs significantly increased the autoaggregation. **(B)** The bar graph shows the percentage of glucan synthesis after MEPB treatment. The results indicate the substantial reduction in glucan synthesis Error bars and asterisks “*,” “**,” and “ns” denote the standard deviation and statistical significance (*p* ≤0.05, *p* <0.01, and “non-significant,” respectively).

Autoaggregation, a phenomenon wherein bacteria interact with one another and settle at the bottom of suspension, is closely associated with biofilm formation. In order to determine the influence of MEPB on the autoaggregation attribute of *S. mutans*, cell suspensions were treated with sub-MICs of MEPB and compared to controls. The outcome revealed that MEPB-treated cells aggregated faster in a concentration-dependent manner. Specifically, cells treated with 128 µg/mL MEPB (sub-MIC) exhibited 80% aggregation, whereas control group demonstrated only 45% autoaggregation after incubation in static condition (*p <*0.01) (**Figure 6A**).

### 3.9 EPS measurement

The effect of MEPB at sub-MICs on the synthesis of water-soluble and insoluble glucans in *S. mutans* was investigated using phenol-sulfuric acid method. The results of EPS quantification divulged that the formation of both water-soluble and -insoluble glucans decreased consistently as the concentration of MEPB increased. Specifically, at 128 µg/mL, MEPB significantly reduced the formation of water-soluble and -insoluble glucans to approximately 52% (*p ≤*0.05) & 85% (*p <*0.01), respectively, as indicated in **Figure 6B**. Interestingly, it was found that the reduction of water -insoluble glucan production was more significant even at the lowest concentration of MEPB inhibited the production of water -insoluble glucan production by 45% (*p ≤*0.05).

### 3.10 Acidogenicity

**Figure 7:**
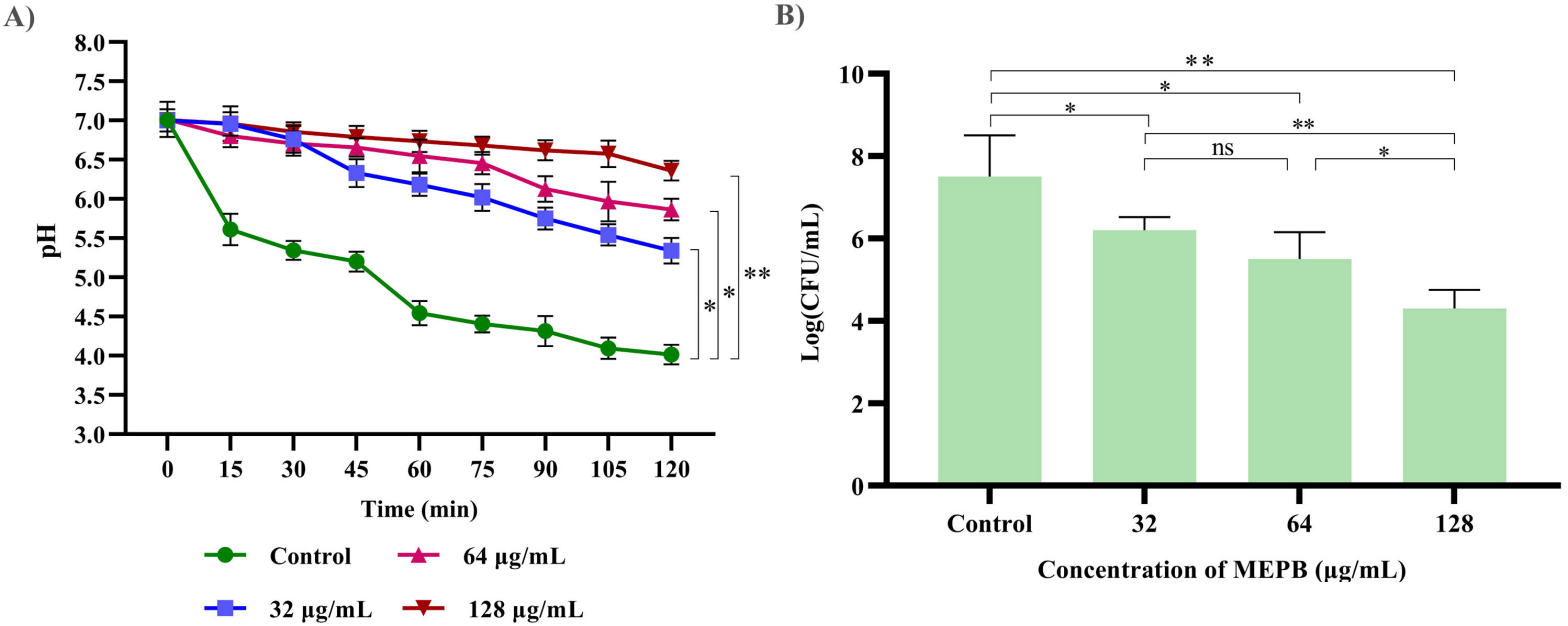
The impact of MEPB on *S. mutans*’s aciduric and acidogenic properties. **(A)** MEPB’s effect on the glycolytic pathway; a decrease in glucose metabolism leads to a reduction in the generation of acid. **(B)** MEPB’s impact on *S. mutans*’s sub-MIC acid tolerance. When compared to untreated controls, MEPB dramatically reduced the survivability of *S. mutans* cells in an acidic environment. Standard deviation and statistical significance (*p* ≤0.05, *p* <0.01 and “non-significant” respectively) are shown by error bars and asterisks “*” “**” and “ns”.

The impact of MEPB at sub-MICs on acid production was assessed by monitoring the pH drop in the *S. mutans* during glycolysis. As depicted in **Figure 7A**, MEPB at 32, 64 and 128 μg/mL reduced the rate of pH drop, in comparison with untreated control (*p* ≤0.05). Furthermore, the terminal pH of the MEPB treatment was significantly higher at 128 μg/mL, very near to pH 6.5 (*p ≤*0.05), than the untreated control, which had a terminal pH of 4. However, there were no considerable divergence in terminal pH values observed at 32 and 64 µg/mL of MEPB when compared with control. This signifies that MEPB at 128 µg/mL have greater potency to interfere with the glycolytic process of *S. mutans*.

### 3.11 Aciduricity

The resilience of *S. mutans* to tolerate the acidic condition under the influence of MEPB at sub-MIC levels was assessed in acidic condition (pH 5.0). When compared to the control group, treatment with MEPB resulted in fewer bacterial colonies. Additionally, MEPB significantly reduced *S. mutans*’s capacity to tolerate acid at 32 (*p ≤*0.05) & 64 μg/mL (*p ≤*0.05) (**Figure 7B**). When compared with control, MEPB (128 μg/mL) significantly reduced survival rate of the cells at a pH of 5.0 by 4-fold log reduction (*p <*0.01).

### 3.12 eDNA extraction

**Figure 8:**
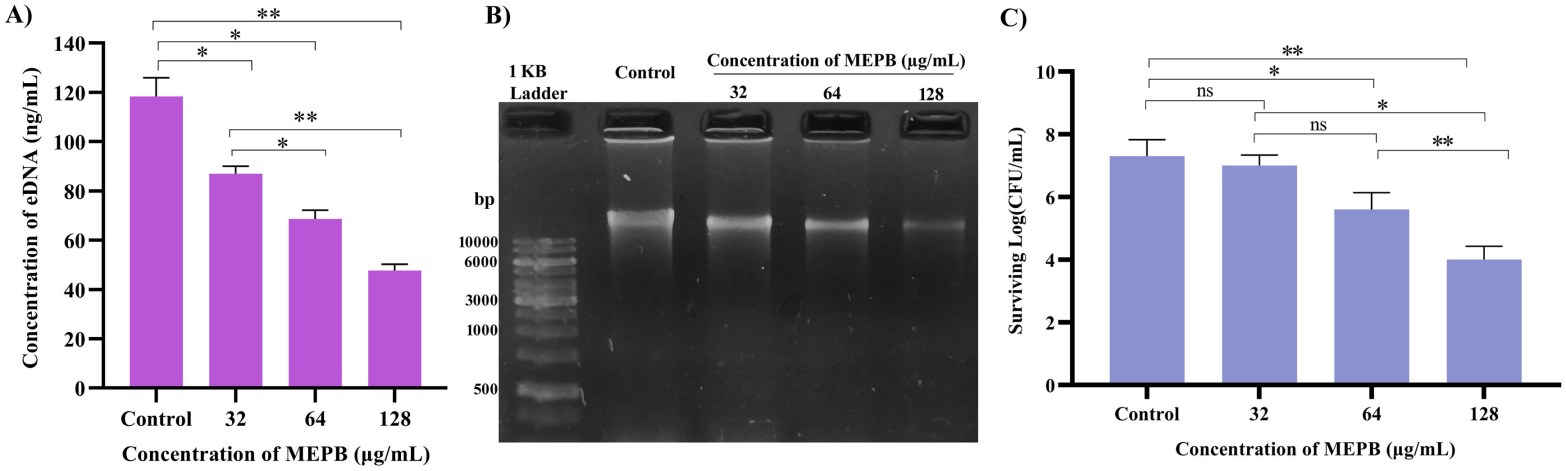
Influence of MEPB on eDNA and sensitivity of *S. mutans*. **(A)** The bar graph represents the decreased amount of eDNA in the biofilm matrix treated with MEPB. **(B)** The representative agarose gel image of eDNA. **(C)**The bar graph indicates reduced surviving ability under H_2_O_2_ during MEPB treatment. Error bars and asterisks “*”, “**”, and “ns” indicate the standard deviation and statistical significance (*p* ≤0.05, *p* <0.01, and “non-significant,” respectively).

To measure the amount of eDNA released by *S. mutans* was determined during and presence and absence of MEPB at sub-MICs. As illustrated in **Figure 8A**, at low MEPB (32 μg/mL), the release of eDNA from *S. mutans* biofilms was dramatically reduced. Treatment with 64 and 128 μg/mL MEPB reduced eDNA leakage by 40% (*p ≤*0.05) and 60% (*p <*0.01) when compared with untreated control. These results were further validated through agarose gel electrophoresis, revealing a significant reduction of band intensity in the increasing concentrations of MEPB treatment (**Figure 8B**).

### 3.13 Effect of MEPB on H_2_O_2_ sensitivity of *S. mutans*

In general, *S. mutans* has the ability to cope the oxidative stress by various protective mechanisms. In order to determine whether MEPB increases *S. mutans’* susceptibility to oxidative stress, it was exposed to H_2_O_2_. The findings revealed that MEPB at 128 μg/mL markedly increased the sensitivity of *S. mutans* to H_2_O_2_ by decreasing the count of viable cells when compared with control cells (**Figure 8C**). These results employ that MEPB possess greater potency to sensitize the *S. mutans* to oxidative stress.

### 3.14 Assessment of gene expression

**Figure 9:**
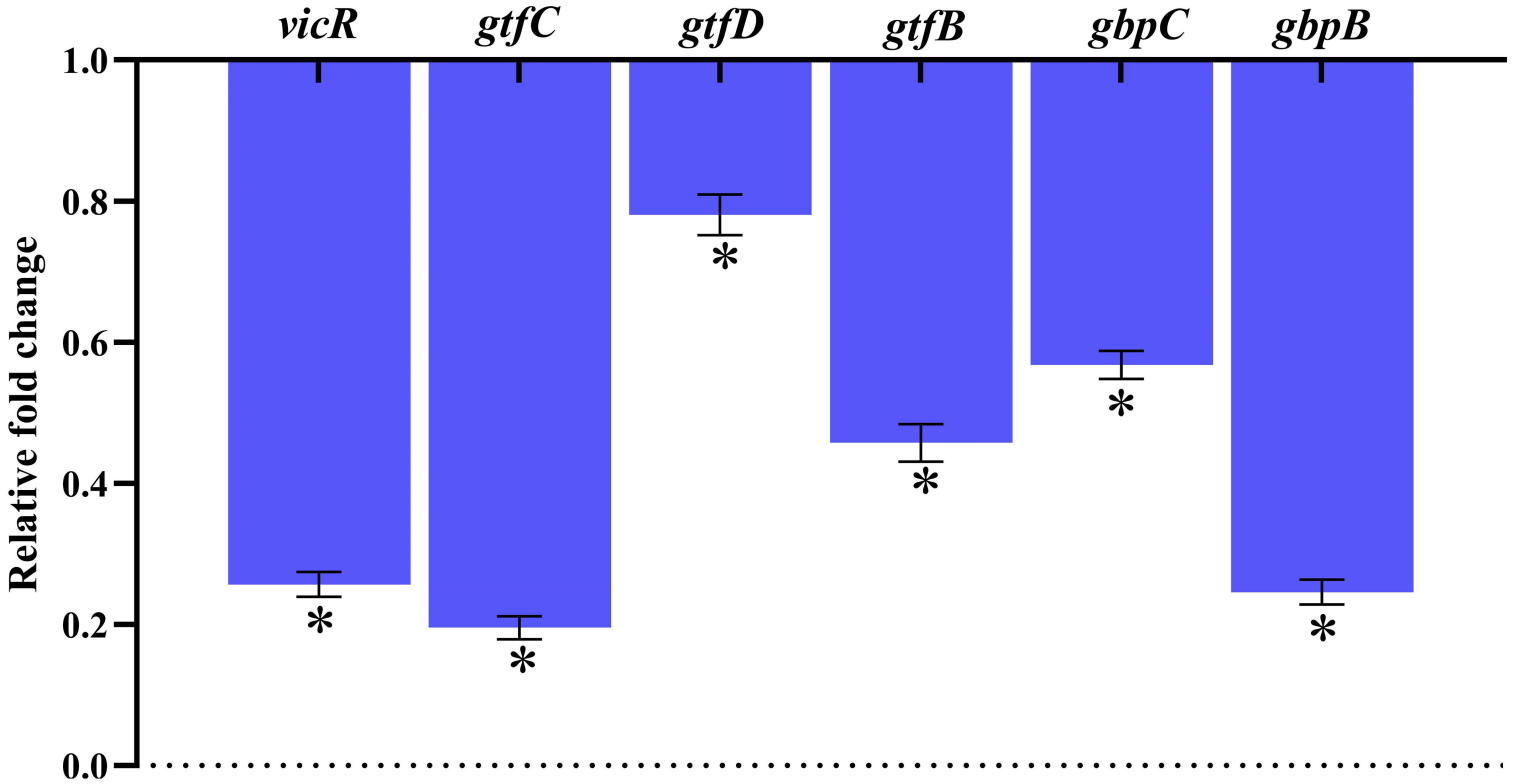
Gene expression analysis. The results of qPCR analysis demonstrate that MEPB negatively regulates the expression of genes linked to *S. mutans* pathogenicity and biofilm formation. The SD and statistical significance (*p ≤*0.05) are shown by error bars and an asterisk (*), respectively.

Real-time RT-PCR was used to evaluate the changes in gene expression of TCS (*vicR*), extracellular polysaccharide synthesis (*gtfB, gtfC* & *gtfD*), adhesion and biofilm formation (*gbpB* & *gbpC*), and TCS in *S. mutans* treated with 128 μg/mL of MEPB. **Figure 9** illustrates that, following MEPB exposure, all assessed virulence genes were substantially downregulated in comparison to the control group *(p < *0.05). The examined genes (*gtfB, gtfC* and *gtfD*) that were exposed to 128 μg/mL MEPB showed a reduction in expression levels of 0.54, 0.198, and 0.82-fold, respectively. Additionally, after being treated with 128 μg/mL MEPB, the expression of genes like *vicR, gbpB* and *gbpC* decreased by 0.25, 0.22, and 0.57fold, respectively.

### 3.15 *In vitro* efficacy of MEPB

**Figure 10:**
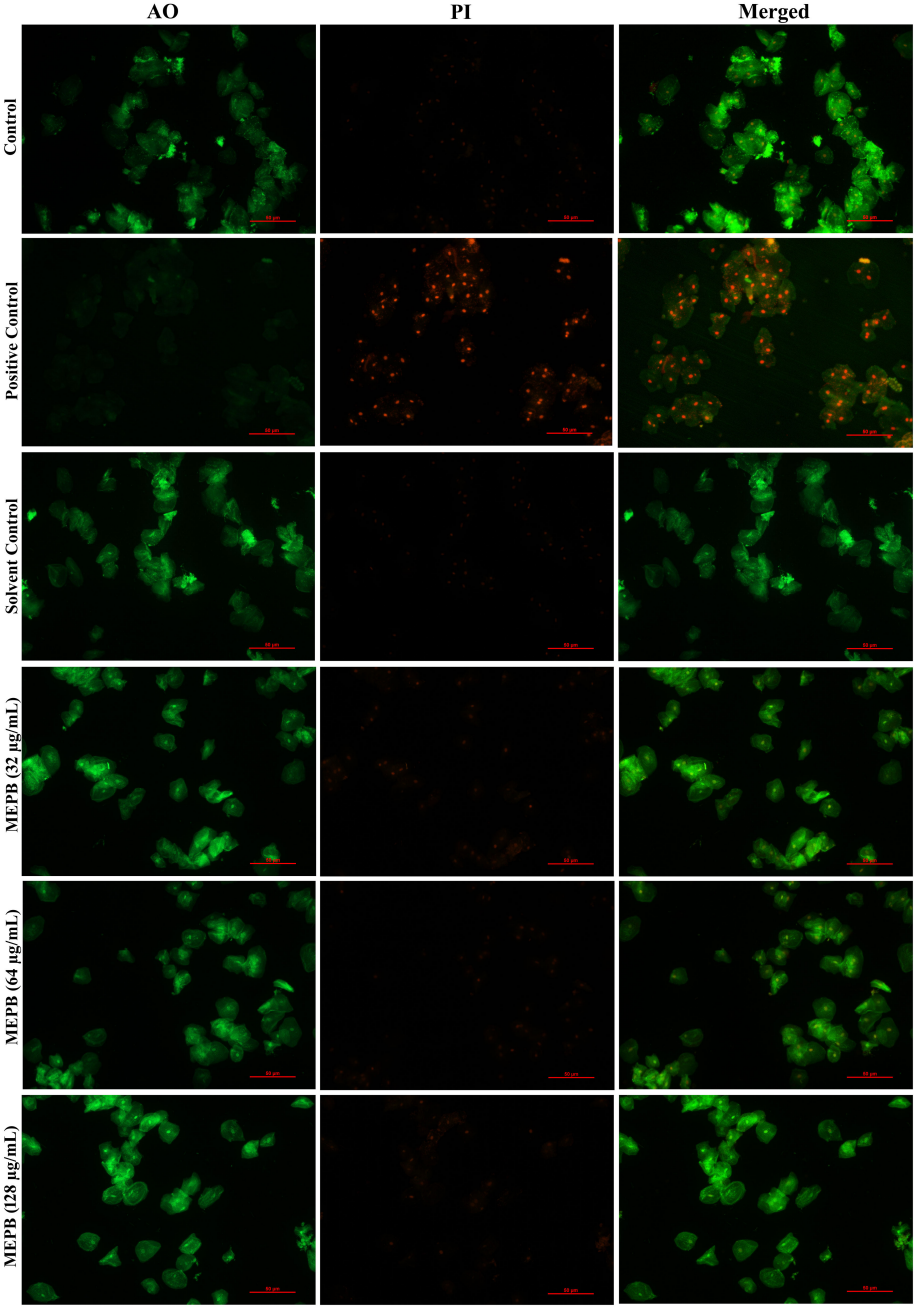
Analyzing the toxicity of human buccal epithelial cells using live/dead staining. The green color in the combined control fluorescence micrographs indicates the existence of living cells. Similarly, a different concentration of the MEPB treatment also revealed the existence of living cells.

The results of toxicity analysis revealed that at the used concentrations MEPB did not exert any toxic effect on HBECs as ascertained through LIVE/DEAD staining with AO and PI. The merged micrograph of the MEPB treated cells indicated the presence of live over dead cells predominantly stained in green color, which is similar to that of control. Whereas the positive control treated with 10% H_2_O_2_ exhibits, killing effect on HEBCs as the presence of dead cells stained in red color (**Figure 10**). The cells treated with MEPB were healthy and normal as well as shows similar kind of morphology as like control cells.

### 3.16 Identification of bioactive compounds in MEPB

**Figure 11:**
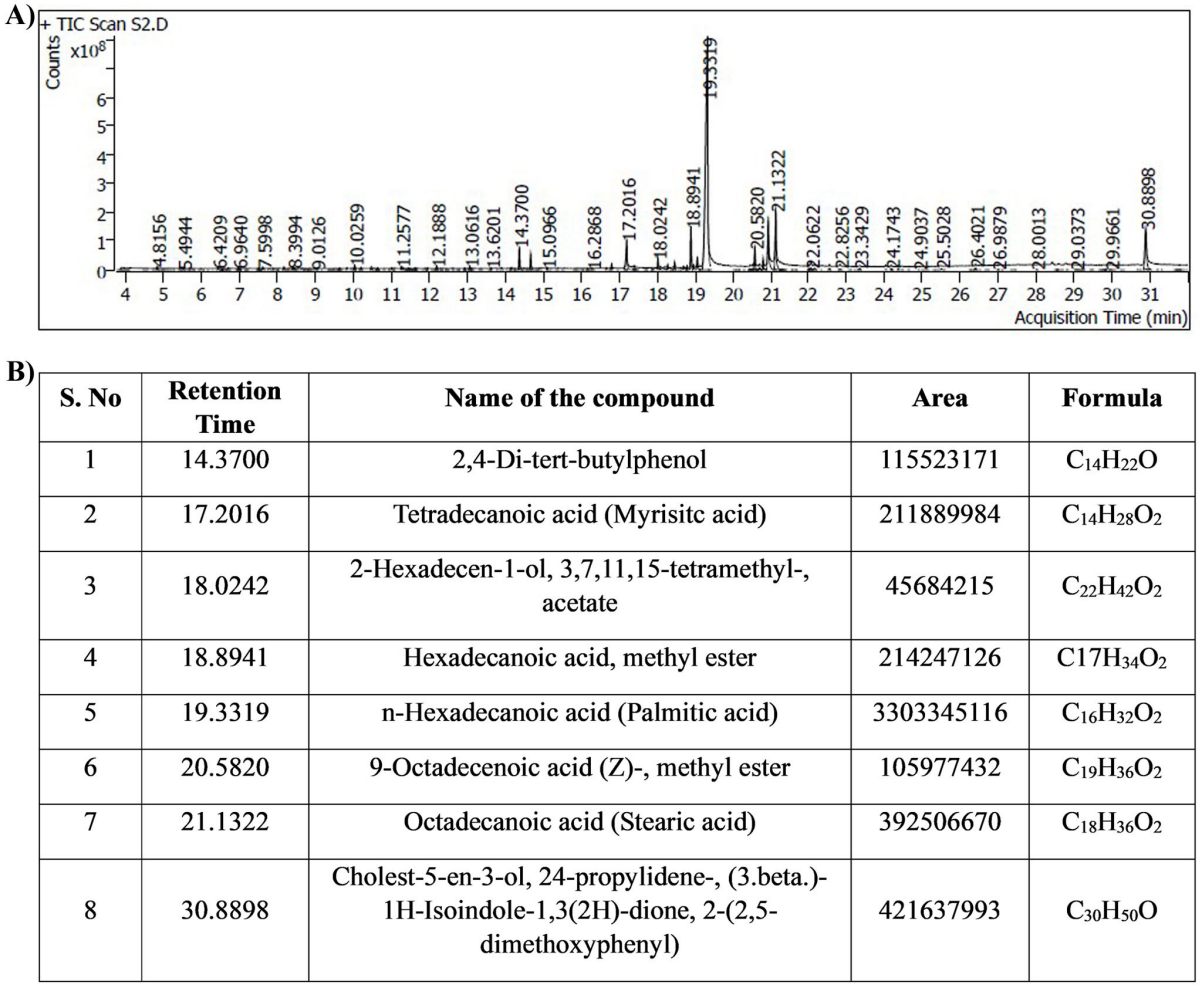
Results of GC-MS/MS analysis. **(A)** Chromatogram displays the peaks of identified compounds. **(B)** The representative table indicated the retention time, compound name, area and molecular formula of identified compounds.

The bioactive compounds from the MEPB were identified through GC–MS/MS technique. The GC–MS/MS analysis indicates that MEPB the presence nine major compounds as depicted in (**Figure 11A**). The eight hits of MEPB are 2,4-Di-tert-butylphenol, Tetradecanoic acid (Myrisitc acid), 2-Hexadecen-1-ol, 3,7,11,15-tetramethyl-, acetate, Hexadecanoic acid, methyl ester, n-Hexadecanoic acid, 9-Octadecenoic acid (Z)-, methyl ester, Octadecanoic acid, Cholest-5-en-3-ol, 24-propylidene-, (3.beta.)- 1H-Isoindole-1,3(2H)-dione, 2-(2,5- dimethoxyphenyl)(**Figure 11B**). Despite the identification of eight hits, the top three hits with highest peak area have been taken for further experiment to identify their antibiofilm potential.

### 3.17 Antibiofilm activity of active compounds

**Figure 12:**
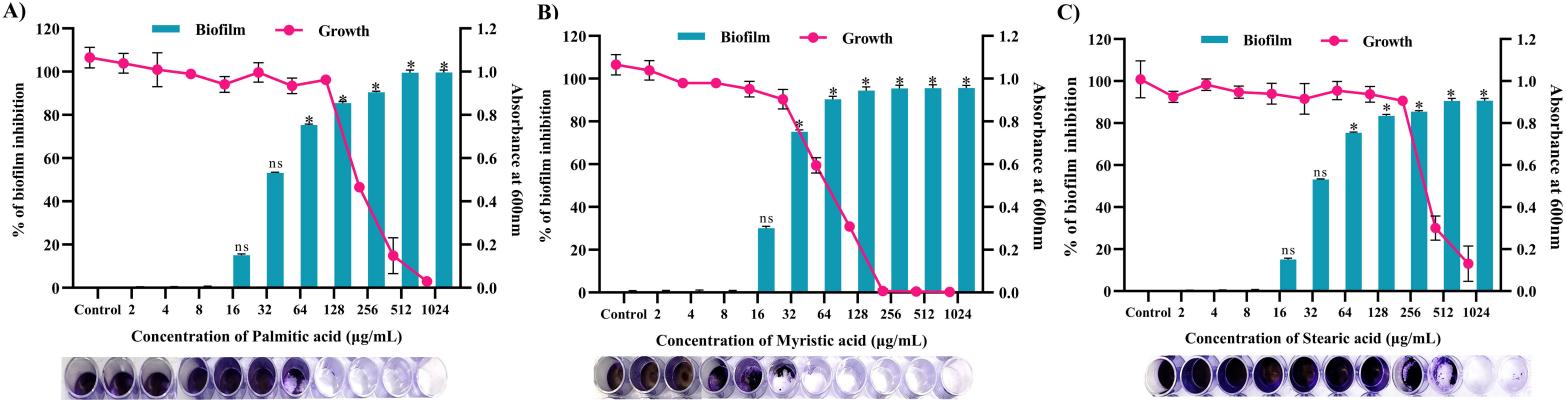
Antibiofilm activity of identified active compounds **(A)** Palmitic acid **(B)** Myristic acid and **(C)** Stearic acid. The bar graph shows the biofilm inhibitory potential of identified hit. Whereas, line indicates the growth OD at used concentrations of identified hits. The standard deviation and statistical significance (*p* ≤0.05, *p* <0.01, and “non-significant”) are denoted by error bars and the asterisks “*”, and “ns”, respectively.

Through a crystal violet-based biofilm biomass quantification method, it was found that palmitic acid displayed the highest antibiofilm activity, inhibiting approximately 85% (*p ≤*0.05) of biofilm formation in 128 μg/mL (**Figure 12A**), followed by myristic acid with over 72% (*p* ≤ 0.05) (**Figure 11A**) inhibition in 32 μg/mL (**Figure 12B**), stearic acid and with more than 83% (*p ≤*0.05) inhibition in 256 μg/mL (**Figure 12C**). Further, these biofilm inhibitory concentrations of all the three fatty acids exhibits no growth reduction as ascertained through broth micro dilution method. Collectively, all three fatty acids contribute to the antibiofilm activity of MEPB.

## 4 Discussion

*S. mutans* is widely acknowledged as the key pathogen in the initiation of dental caries owing to its distinctive virulence factors and significant presence in oral biofilm (Moye et al., 2014). Recent studies have focused on utilizing the anticaries agents produced from medicinal plants, as well as common antimicrobials including CHX, NaF and triclosan (Li et al., 2020). These agents have been extensively studied for their propensity to inhibit biofilm formation and caries progression. However, traditional anticaries agents primarily reduce the viability of biofilm cells rather than its pathogenicity, leading to adverse consequence (Selvaraj et al., 2020). This underscores the urgent need for alternative and innovative therapeutic approaches to mitigate biofilm-related illnesses, particularly in oral cavity. In response to this need, the present study has explored anti-virulence strategy that interrupts and target specific pathogenic pathways, without compromising its viability (Prasath et al., 2019). This approach aims to prevent the development of resistance and reduce the negative side effects associated with traditional antimicrobial treatments. To address these drawbacks, utilizing marine macroalgal sources presents a promising approach, as they contain various biomolecules effective against diverse microorganisms. Notably, there have been no studies or reports divulging the anti-virulence propensity of *P. boergesenii* extract on *S. mutans*.

Primarily, the influence of MEPB on the viability of planktonic cells of *S. mutans* was evaluated. The results clearly indicate that MEPB exhibits an antibacterial effect at 1024 μg/mL with growth inhibition of about 95%. Thus, the study found that 1024 μg/mL of MEPB as MIC against *S. mutans* (**Figure 1**). Ideally, an anti-virulence agent should not influence the growth and viability of the pathogen, as antibacterial agents impose enduring stress on bacteria, leading to the emergence of resistance (Kasthuri et al., 2022). To address this, the bactericidal efficacy of MEPB at sub-MICs of was assessed through growth curve and metabolic viability assay. The finding from the growth curve analysis (**Figure 2A**) indicate that no apparent difference in the growth pattern of MEPB-treated and untreated *S. mutans* at sub-MICs. This observation was corroborated by the alamar blue assay results (**Figure 2B**), confirming that MEPB at sub-MICs did not exhibit bactericidal activity, indicating that none of the test concentrations used had any effect on cell growth or survival. Methanol up to 10 µL/mL was used as vehicle control showed no impact on the physiology or behavior of *S. mutans*, the minimal volumes used in the study—0.64, 1.28, and 2.56 µL—are unlikely to cause any adverse effects. Therefore, the observed anticariogenic efficacy of MEPB can be attributed solely due to active compounds of MEPB, not methanol which is used as a solvent. Consequently, with such low concentrations of methanol, the likelihood of solvent-induced lethality is significantly minimized.

The present study demonstrated that MEPB is an effective antibiofilm agent. MEPB was able to completely prevent *S. mutans* biofilm formation *in vitro*, after 24 h incubation. The BIC of MEPB was 128 μg/mL, as this concentration reduced more than 90% biofilm rather than other sub-MICs (**Figure 3**). Light microscopic observation (**Figure 4A**) of biofilms corroborated findings from the biofilm quantification assay (**Figure 4B**), showing a notable reduction in surface area covered, and density and biofilm biomass in treated groups. The results of CLSM micrographs of *S. mutans* biofilms treated with MEPB revealed no impact on cell viability (**Figure 4C**), with control and treated samples displaying a higher proportion of live cells than dead cells. This underscores MEPB’s efficacy in reducing *S. mutans* biofilm formation without hindering bacterial growth.

Ability to adhere to the tooth surface has been recognized as the crucial step in dental caries formation (Viszwapriya et al., 2017). In general, there are two different mechanism of adherence was adopted by *S. mutans* namely, sucrose -dependent and -independent mechanisms. Several studies have consistently highlighted the pivotal role of sucrose dependent adherence in both in initial attachment and subsequent biofilm formation over sucrose independent adherence. A considerable reduction in sucrose-dependent and -independent adherence was seen in this study by MEPB, with a reduction of 75% (*p* <0.01) and 65% (*p* ≤0.05), respectively (**Figure 5A**).

Cell surface hydrophobicity is an important element in host-pathogen interactions and the adhesion of bacteria to tooth surfaces through hydrophobic interactions (He et al., 2019). Prior research suggests that limiting the hydrophobic nature of *S. mutans* inhibits the bacterial attachment subsequent biofilm formation and caries development (Vijayakumar and MuhilVannan, 2021). In the present study, MEPB at sub-MICs demonstrated a significant reduction in the percentage of hydrophobicity indicating that MEPB has the ability to amend the hydrophobic characteristics of *S. mutans* (**Figure 5B**).

Bacterial biofilms are composed of a complex matrix that includes proteins, extracellular polysaccharides, lipids and eDNA. While all these components are crucial for biofilm formation, the EPS is particularly significant as it confers resilience to the biofilm, providing protection against antimicrobial agents. In general, EPS of *S. mutans* encompasses two different categories of glucans such as water -soluble and -insoluble glucans that contribute to the framework of the EPS matrix and overall structure of the biofilm (Xiao et al., 2012). In this, MEPB at 128 µg/mL drastically reduced both water-soluble and -insoluble glucans production (**Figure 6B**). This significant reduction in glucan production by MEPB highlights its efficacy in inhibiting biofilm formation via anti-adherence mechanisms as the glucans serves as primer for adherence of *S. mutans* to the tooth surface.

The physiological traits of *S. mutans*, such as its capacity to produce acid and withstand low pH, are essential for the tooth demineralization and subsequent caries progression (Moye et al., 2014). These acids are primarily produced through the glycolytic process, as *S. mutans* solely depend on glycolysis for energy production. Maintaining the pH range of 5.0–5.5 is essential for balancing the processes of tooth enamel demineralization and remineralization. When the pH of the surrounding environment falls below this critical threshold due to acid accumulation, tooth demineralization can occur, leading to the initiation of dental caries. Our findings show that increasing MEPB concentrations caused a progressive decrease in the initial rate of pH decline, culminating in final pH values that exceeded the crucial pH threshold (**Figure 7A**). These findings show that MEPB reduces acidogenicity, possibly by interfering with the activity of glycolytic enzymes involved in acid generation, and therefore avoiding tooth demineralization. Acid tolerance is another one of the important traits of *S. mutans* connected with cariogenic potential (Matsui and Cvitkovitch, 2010). Our results show that MEPB reduces bacteria viability at pH 5.0 (**Figure 7B**). Furthermore, the results show that MEPB has a significant influence on acid production and tolerance.

To further understand MEPB’s antivirulence activity on *S. mutans* and to assess its influence at transcriptional level, we assessed the expression levels of potential genes involved in biofilm formation and aggregation. Previous studies emphasizes that the expression patterns of *gtfB* and *gtfC* were similar, as these genes are co-transcribed and co-regulated, being located in the same locus. In contrast, *gtfD* is located and regulated separately from the *gtfBC* locus (Zhang et al., 2021). Previous research has shown that in the availability of sucrose the expression of *gtf* genes was enhanced (Duarte et al., 2008). Interestingly, MEPB successfully suppressed the expression of these *gtf* genes even in the presence of sucrose, a finding supported by the significant decrease in glucan synthesis. This implies that MEPB has a great deal of promise as an anti-virulence agent even in the sucrose rich diet conditions. Further, reports have demonstrated that downregulation of *gbpB* and *gpbC* clearly altered the initial steps of sucrose-dependent biofilm formation (Duque et al., 2011). In alignment with this finding, our gene expression analysis also suggested the downregulation of *gbpB* and *gpbC*, which inhibits sucrose dependent adherence (**Figure 9**).

Two-component regulatory systems (TCS) are essential for regulating the expression of *S. mutans* virulence genes involved in biofilm formation, adaptability, survival and virulence production (Stipp et al., 2013). Among TCS, VicRK is a key signal transduction pathway where VicR is a global response regulator (RR) and VicK functions as a histidine protein kinase (Wang et al., 2021). According to the reports, the VicRK signal transduction system regulates the genes (*gtfBCD*) and biofilm development that has been consistent with the current study, which found that MEPB treatment resulted in *vicR* downregulation and a reduction in reduced glucan production and biofilm formation. In addition, *vicR* known to regulate the expression *gbpB* and *gpbC*, the mutant of *vicR* demonstrates reduced expression of both *gbpB* and *gpbC* (Biswas et al., 2007).

Research indicates that eDNA can improve *S. mutans*’ adherence to hydrophobic surfaces, which in turn increases the formation of biofilm (Klein et al., 2015). Furthermore, research found that increased eDNA release in the supernatant was seen with *vicR* deletion (Stipp et al., 2013). Surprisingly, in contrast to the eDNA from the control, the eDNA content in the MEPB treated biofilm was totally suppressed (**Figures 8A, B**). The increased release of eDNA material into the outside environment may be the cause of reduced eDNA content.

Moreover, our study showed that MEPB treatment dramatically changes *S. mutans*’ growth pattern in liquid media. The MEPB-treated *S. mutans* cells showed aggregation and settled at the bottom (**Figure 6A**), a behavior also seen in *vicK* mutant strains (Senadheera et al., 2005), in contrast to the control cell suspensions of *S. mutans*, which expanded uniformly over night. According to Tremblay et al. (2009), the *vicRK* genes are co-regulated in an operon model, suggesting that downregulating *vicR* might also reduce *vicK* expression and cause cell aggregation in response to MEPB treatment. Further, these results were well corroborated with the finding of Viszwapriya et al. (2017) as well as Vijayakumar and MuhilVannan (2021). Additionally, it is known that the *vicK* knockout mutant is more H_2_O_2_ than the wild-type. Our findings also demonstrate that MEPB treatment significantly sensitize the *S. mutans* cells to the H_2_O_2_ (**Figure 8C**). Altogether, the downregulation of *vicRK* leads reduced the biofilm and virulence factors through downregulation of *gtf*s and *gpb*s (**Figure 9**). This finding is consistent with earlier studies where betulin (Viszwapriya et al., 2017) and 3,5- di tert butylphenol (Vijayakumar and MuhilVannan, 2021) was found to inhibit *S. mutans* biofilm formation by targeting *vicR.*

To ensure suitability for therapeutic use, assessing the cytotoxicity of the bioactive compound is crucial. Using the LIVE/DEAD staining technique, we evaluated the influence of MEPB on the HBECs viability (**Figure 10**). The results clearly demonstrate the absence of any cytotoxic effects of MEPB on HBECs, indicating its potential application of MEPB is safe to be incorporated as therapeutic agent to prevent/treat dental caries.

In this investigation, GC-MS/MS analysis was employed to discern the active constituents of MEPB, uncovering prominent bioactive compounds. These findings corroborate previous studies indicating the abundance of fatty acids in *P. boergesenii* (Kalasariya et al., 2023). Totally eight hits were obtained from the GC-MS/MS as depicted in **Figure 11B**. On the basis of peak area percentage and retention time, three different fatty acids were selected such as palmitic acid, myristic acid and stearic acid for further *in vitro* antibiofilm assay.

Recent literatures evidenced that fatty acids have significant antibiofilm potential, quite a lot of fatty acids have been documented to specifically inhibit or disrupt biofilm formation by numerous pathogenic microorganisms, such as *Staphylococcus aureus* (Davies and Marques, 2009), *Pseudomonas aeruginosa* (Wenderska et al., 2011) and *Candida albicans* (Prasath et al., 2019). Nevertheless, only a few researches have demonstrated the antibacterial and antibiofilm properties of fatty acids against *S. mutans*. For example, Huang et al. (2011) revealed that n-6, n-7, and n-9 FAs and their esters had antibacterial activity against oral microorganisms. Moreover, it has been demonstrated that oleic and linoleic derived from *Withania somnifera* have been demonstrated to efficiently reduce EPS and acid generation rate in biofilms while preserving bacterial viability (Pandit et al., 2015). Furthermore, it has been discovered that the oleic acid promotes the EPS reduction activity of fluoride in *S. mutans* biofilms, hence reducing the fluoride concentration without hampering the survivability of biofilm (Cai et al., 2015). The fatty acids namely, as palmitic acid, myristic acid and stearic acid identified in the current study fall under the category of saturated fatty acids, widely found in many of the living organism. According to Liu et al. (2012), all three of the fatty acids have been shown to reduce *Proteus mirabilis*’s swarming motility in a concentration-dependent manner. In a similar vein, downregulating QS-related genes including *luxS* and *luxR* after palmitic acid treatment of *Vibrio* spp. cells hindered EPS synthesis and biofilm formation (Santhakumari et al., 2017).

Furthermore, intracellular fatty acid arrays rich in palmitic, myristic and stearic acid derived from endophytic *Arthrographis kalrae* inhibited the development of biofilms and EPS production (Abdel-Aziz et al., 2020). In line with this finding in the current study, the MEPB and its active components (palmitic acid, myristic acid and stearic acid) inhibited the biofilm and virulence traits of *S. mutans*, without posing any antibacterial effect, emphasizes that these fatty acids hold a great potential to be used in dental care applications (**Figure 12**). Meanwhile, further investigations are needed to completely understand the anticariogenic potential of the identified fatty acids for dental caries prevention treatment.

## 5 Conclusion

This study demonstrates that MEPB, at sub-MIC levels, effectively inhibits several cariogenic virulence traits of *S. mutans*, including acid production & tolerance, biofilm formation, EPS production and expression of virulence genes. These findings suggest that MEPB could serve as a novel marine source to prevent/control the development of dental caries. Furthermore, GC-MS/MS analysis has identified the active compounds in MEPB that are effective against biofilm formation. Due to its non-toxic and anti-infective properties, MEPB shows promise as a therapeutic agent targeting *S. mutans* by disrupting its virulence without affecting bacterial viability. Incorporating MEPB/active compounds into dentifrices could be an viable strategy for the prevention and treatment of dental caries.

## Data Availability

The original contributions presented in the study are included in the article/supplementary material. Further inquiries can be directed to the corresponding author.
